# Supplementary material for: Crystal growth in confinement
Source: Nat Commun. 2022 Nov 16;13:6990. doi: 10.1038/s41467-022-34330-5 (PMC9669051; doi:10.1038/s41467-022-34330-5)
Supplement: Supplementary file 1 — Supplementary Information [file 41467_2022_34330_MOESM1_ESM.pdf]

Supporting Information for Crystal growth in confinement

Felix Kohler,[1] Olivier Pierre-Louis,[2] and Dag Kristian Dysthe[1]
[1] *The NJORD Centre, Dept of Physics, University of Oslo, P.O.box 1048 Blindern, 0316 Oslo, Norway*
[2] *Institut Lumière Matière, UMR5306 Université Lyon 1 - CNRS, 69622 Villeurbanne, France*

This PDF file includes:
Supplementary Text
Figs. S1 to S6
Other Supplementary Materials for this manuscript include the following:
Supplementary Movies 1-6

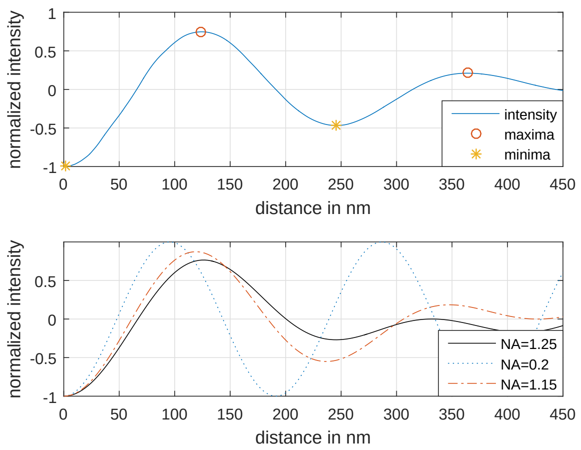

FIG. S1. Intensity of the interference term as a function of the distance d to the glass a) Reference measurement performed with a spherical lens ($r = 7.7mm$) b) Intensity as a function of d for different effective numerical apertures calculated using the equations in the text.

ESTIMATION OF DISJOINING PRESSURE IN CRYSTAL-SPACER CONTACTS

When two surfaces approach each other the liquid film between the surfaces can support a normal stress without being squeezed out [1]. This is termed the *disjoining pressure* of the fluid film on the solid surfaces. In the nanometer range where the disjoining pressure is probed in our experiments, van der Waals forces are negligible. The electric double layer contribution dominates and scales with the Debye length and at small fluid film thickness (1-5 molecular layers) there are so-called steric repulsive forces that increase rapidly with decreasing distance z. In order to squeeze out the last 2-3 molecular layers of water, pressures of the order 1 GPa are required [1]. Thus at high ion concentration one expects the fluid film thickness z to be in the range 0.8-1.6 nm for all pressures in the range 0.1 MPa to 1 GPa. Even though the liquid can support a normal pressure it still behaves like a fluid in the sense that diffusion is almost as fast as in bulk until the liquid film thickness z is reduced to 2 molecular layers [2].

In our experiments the growing crystals are resting on small spacers that keep the distance ζ between the growing crystal and the supporting glass surface between 10 and 130 nm. If there were no spacers the equilibrium distance would be of the order 1 nm. This would make the optical contrast of the RICM technique too low to accurately measure height differences and the rate of ion transport by diffusion would be a factor 30-1000 lower, thus halting the process we wish to study. Figure S2 shows the distribution and height of spacers measured by AFM, the change of ζ when a molecular layer is grown and the relaxation back to the equilibrium distance and an illustration of the step flow through the contact between a spacer and the crystal. The relaxation back to the equilibrium distance demonstrates clearly that the crystal is supported by the disjoining pressure in the fluid film in the contacts with the spacers.

The contact (disjoining) pressure can be estimated by noting that a cap of about 1 nm height of the spacer will contribute as support for the fluid film. Thus for an average spacer size of assumed spherical shape of diameter 20 nm the supporting area is $A_{cont} = \pi(20 \sin \arccos 19/20)^2 \approx 10^{-16}$ m^2. The $NaClO_3$ crystals of linear size L are typically half as high as wide, thus their volume are $V = L^3/2$. The density difference of the crystal and the saturated solution is $\Delta\rho$ =1100 kg/m^3. The equilibrium pressure in the contact can then be estimated as

$$p_0 = \frac{\Delta\rho g V}{2N_{cont}A_{cont}} = \frac{1}{2}\Delta\rho L g \alpha = 5500 L\alpha, \qquad (1)$$

where $\alpha = A_{cryst}/N_{cont}A_{cont}$ is the ratio of crystal area to contact area. This ratio can be estimated from Figure S2 B where $A_{cryst} = (10\mu\text{m})^2 = 10^{-10}$ m^2 and there are 1848 spacer particles. The real, unknown number of contacts $3 < N_{cont} < 1800$ depends on the size distribution and stiffness of the spacer particles. The area ratio is then $\alpha = A_{cryst}/N_{cont}A_{cont} \approx 10^6/N_{cont} \approx 550 - 3.3 \cdot 10^5$. It follows that for a typical crystal size used in our experiments, $L = 100\mu$m the disjoining pressure in the spacer-crystal contacts are in the range $300 - 2 \cdot 10^5$ Pa. This indicates that even if the crystal is supported by only 3 spacer particles the contact pressure p_0 is only about one atmosphere, well below the pressure necessary to squeeze the fluid film thickness to only 1-2 molecular layers.

We may use the relaxation of the fluid film thickness z between the top of particles and the crystal towards the

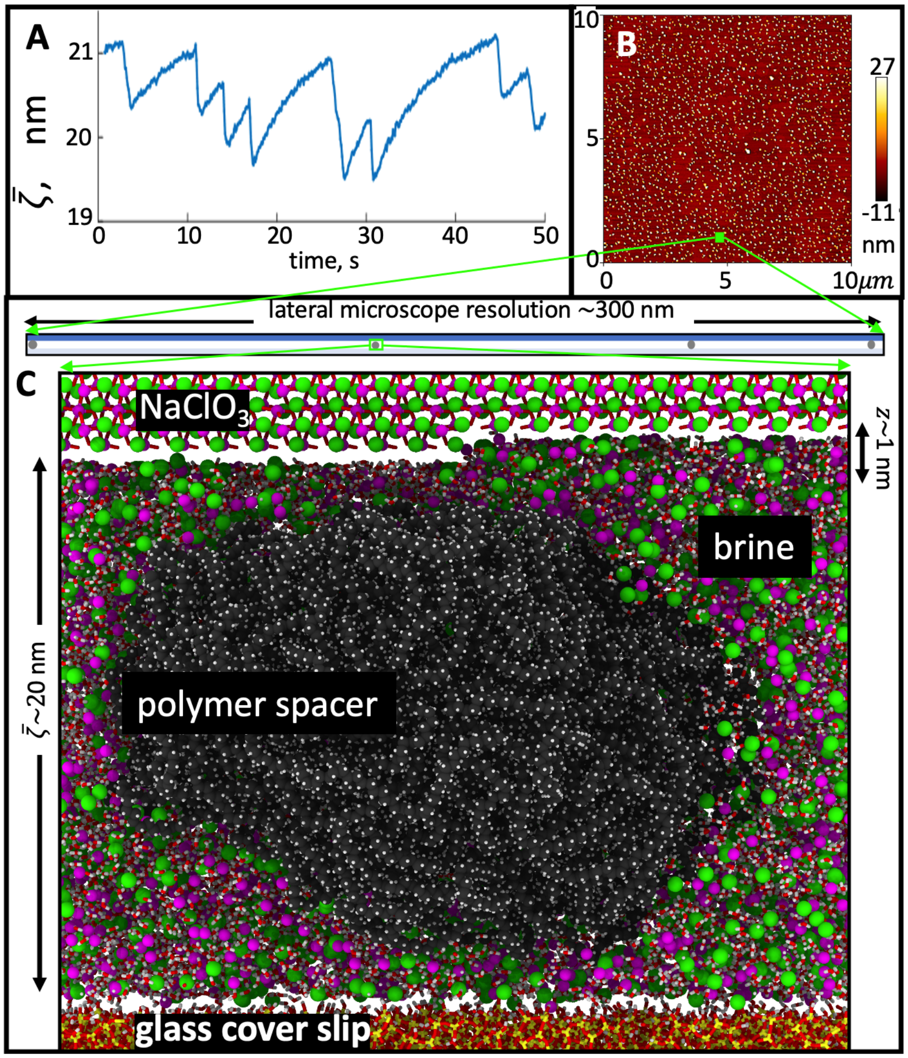

FIG. S2. Step flow through contacts. **A** The average gap distance, $\bar{\zeta}$ between the crystal and the glass support as function of time. The rapid drops by 0.66 nm in $\bar{\zeta}$ correspond to two molecular layers (one unit cell thickness) growing on the crystal in the confined region by step flow. After the gap has been reduced it slowly relaxes towards the equilibrium distance again. **B** Atomic force microscope (AFM) image of glass surface before mounting of cell. The glass support is evenly covered by spacers of radius $r \approx 20$ nm with a spacer density of $\approx 18\mu\mathrm{m}^{-2}$. **C** Vertical cut of crystal, spacers and glass illustrating the crystal step edge propagating through the thin ($\approx$1 nm) liquid film that supports the weight of the crystal. The image illustrates the correct relation between 10 nm spacer size, 0.33 nm step height and $\approx$1 nm fluid film thickness in the contact. The densities of water and ions in crystal and solution are also to scale. When two steps have passed, the liquid layer has become 0.66 nm thinner and the disjoining force in the liquid layer increased. This increased force pushes the crystal back towards equilibrium as observed in **A**.

equilibrium distance z_0 to probe the disjoining pressure further. Growth of a new layer reduces the fluid film thickness by 0.66 nm. Excess disjoining pressure $\Delta p = p - p_0$, pushes the crystal back towards the equilibrium distance, while viscous drag of fluid flowing between the crystal and the support opposes the vertical motion. We may assume that the disjoining pressure, p, at these distances is an exponential function of distance:

$$\Delta p = p - p_0 = p_0(e^{-(z-z_0)/\lambda_D} - 1), \tag{2}$$

where λ_D is the Debye length. Since the crystal moves as a whole, the change of the average distance ζ between the crystal and the glass substrate is equal to the change in the film thickness at the top of particles: $\zeta - \zeta_0 = z - z_0$. A circular plate of radius R at distance ζ from another surface that is subjected to a disjoining pressure Δp at the contacts will have the vertical velocity

$$\begin{aligned} v_z &= \frac{N_{cont} A_{cont} \zeta_0^3}{3\pi\eta R^4} \Delta p & (3)\\ &= \frac{2\zeta_0^3 \Delta\rho g}{3\pi\eta R}(e^{-(\zeta-\zeta_0)/\lambda_D} - 1) & (4)\\ &= v_{z,0}(e^{-(\zeta-\zeta_0)/\lambda_D} - 1), & (5)\end{aligned}$$

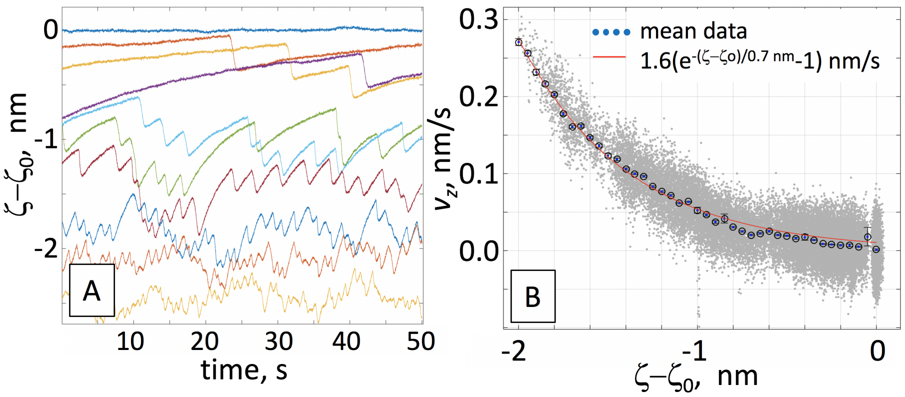

FIG. S3. Relaxation of fluid film distance due to disjoining pressure. **Left:** Mean fluid film thickness minus equilibrium value $\zeta - \zeta_0$ for many different nucleation rates. Growth of a new layer reduces the fluid film thickness by 0.66 nm. Excess disjoining pressure $\Delta p = p - p_0$, where p_0 is the equilibrium pressure pushes the crystal back towards the equilibrium pressure. Viscous drag of fluid flowing in the fluid film to allow the relaxation opposes the vertical motion. **Right:** Vertical velocity of crystal as function of $\zeta - \zeta_0$ from derivative of positions in left subfigure.

where η is the viscosity, and we have used the approximation $\zeta \approx \zeta_0$ in the prefactor. The crystal in the experiment shown in Fig. S3 had $R \approx 50\mu$m and $\zeta_0 \approx 100$ nm. Using $\eta \approx 10^{-3}$ Pa s the predicted velocity is $v_{z,0} \approx 0.2$ nm/s. For a monovalent salt at 7 molar the theoretical Debye length is 0.13 nm and the high concentration decay length has recently found to be 3 nm [3].

In Fig. S3 we show the displacement curves at different nucleation rates and the agglomerated velocity - distance curve with the fit yielding the model parameters $v_{z,0} = 1.6$ nm/s and $\lambda_D = 0.7$ nm. This is reasonably close to the $v_{z,0}$ predicted from viscous drag and the decay length is between the two estimates given above. Based on the fitted Debye length we expect the crystal and weight supporting grains to be separated by only 3-4 layers of adsorbed solution [1]. If the fluid film thickness approached 1 molecular crystal layer one would expect that the step flow could be pinned in the spacer-crystal contacts. In only very few experiments have we observed such pinning of the crystal growth step front by spacer particles.

Nucleation

Standard nucleation theory

The standard theory of nucleation is reported in many books and lecturenotes on crystal growth [4]. In this theory, the free energy of a monolayer island is composed of two contributions. The first one is the chemical potential gain $\Delta\mu$ caused by the crystallization of the ions when the ionic concentration in the liquid exceeds the solubility. The second contribution is the free energy cost of the formation of the atomic step surrounding the monolayer island. Since the first contribution is proportional to the area of the island while the second is proportional to the perimeter, the second term always wins for small islands. As a consequence, there is a free energy barrier that must be surmounted in order to form the two dimensional layer. The passage over this barrier is triggered by random thermal fluctuations. Once the size of the island is larger than the critical size corresponding to the energy barrier, the islands grow irreversibly to decrease their total free energy.

We assume small supersaturations $\sigma \ll 1$, so that the chemical potential reads

$$\Delta\mu = nk_BT\ln(1+\sigma) \approx nk_BT\sigma \qquad (6)$$

where the factor $n = 2$ comes from the presence of two ions in the liquid for one molecule in the solid. If the solution is not ideal, the chemical potential is still proportional to σ for $\sigma \ll 1$, but the factor n can be affected when taking into account the activities of relevant species in the fluid.

Under the assumption of isotropic step properties, the rate of formation of new monolayer islands per unit facet area in the presence of a supersaturation σ reads

$$J = J_c(\sigma)\,\mathrm{e}^{-\sigma_c/\sigma} \qquad (7)$$

where

$$J_c(\sigma) = \frac{\rho_s\bar{\alpha}}{\Omega_2^{1/2}}(n\sigma)^{1/2} \qquad (8)$$

$$\sigma_c = \frac{\pi\Gamma^2}{\Omega_2 n} \qquad (9)$$

We have defined the concentration ρ_s (number per unit area) of initial seeds for nucleation, the orientational average of the kinetic coefficient $\bar{\alpha} = \alpha\langle k(\theta)\rangle$ discussed in the main text, the molecular area Ω_2 in a monolayer (in the case of $NaClO_3$, we have $\Omega_2 = 2z_0^2$ where z_0 is the step height) and $\Gamma = \Omega_2\frac{\gamma}{k_BT}$, where γ is the step free energy per unit length, and k_B is the Boltzmann constant.

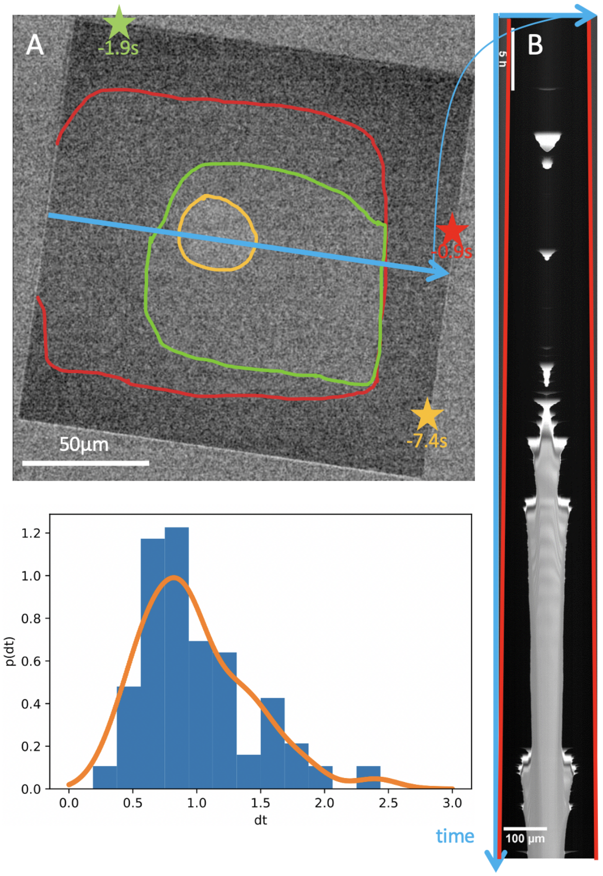

FIG. S4. Fluctuations in nucleation and cavity formation. The high speed and high vertical resolution of our measurement technique allows us to measure the distribution of the nucleation rates as shown in subfigure **A**. Subfigure **B** is the intensity along the blue arrow in subfigure **A** during the entire Movie S3. The crystal size (indicated by red lines) increases continuously with time and finally exceeds a threshold value where the cavity is stable and grows with fluctuations in size similar to the initial fluctuations. The width of the rim (black) outside the cavity (bright) is smaller than half the crystal size during the fluctuating cavity period. This corresponds to an unstable regime between two "phases" (no cavity and cavity) of the nanoconfined system. The fluctuation between cavity and no cavity and fluctuation of the cavity size is due to the fluctuation in time between nucleation of new layers, dt, displayed in subfigure **C**.

The two main conditions of validity of (7) are: (i) $\sigma \ll \sigma_c$, which ensures that the nucleation energy barrier is larger than $k_B T$, and (ii) nothing special occurs at the molecular scale when the nucleus is composed of a small number of molecules, i.e., no extremely stable intermediate molecular configuration and no extremely slow process at the molecular scale.

Localization of nucleation

If the supersaturation is the same everywhere on the facet, then the total nucleation rate is simply JL^2 where L^2 is the area of a square facet of lateral extent L. Assuming that the change of the facet size L is negligible between two nucleation events, as seen in experiments, the nucleation time $\tau_N = 1/(JL^2)$ given by the expression (7) can be fitted to the data (see Figure 2) using two free parameters: σ_c and ρ_s. We then find a surface coverage of initial seeds for nucleation $\Omega_2\rho_s \approx 2.5\times10^{-8}$ where $\Omega_2 = 2z_0^2$ is the area occupied by one molecule in the monolayer. In addition, we obtain $\sigma_c = 1.1 \pm 0.1$, leading to $\Gamma = 0.40 \pm 0.02$nm.

However, in general the supersaturation is not homogeneous on the confined facet. Along the periphery of the facet, the supersaturation is expected to be constant due to continuous exchange of mass with the neighboring bulk phase. However, somewhere on the facet, the supersaturation can be lower due to the growth of some step that have consumed part of the ions in the liquid film. Such a depletion survives even after the passage of a step during a time of the order of $\tau_D = L^2/(4D)$ where L is the lateral size of the facet. Using $D = 0.6 \times 10^{-9}$m^2.s^{-1}, and $L = 175\mu$m (as in Figure 2D), we obtain $\tau_D \approx 10$s. This timescale is of the same order as the time between two nucleation events (from 1 to 100s) in the regime where there is never more than one step on the confined facet. The absence of clear separation between these two timescales prevents a quantitative prediction of the supersaturation profile, which controls the localization of nucleation close to the edge. However, a generic analysis presented below catches the essence of the localization of nucleation.

In order to investigate the influence of supersaturation gradients on the localisation of nucleation events, we consider a simplified one-dimensional geometry, with a straight facet edge, where the supersaturation is fixed to a value σ_+. We also assume that σ decreases monotonously up to a value σ_- at some distance d from the edge. We therefore assume that the supersaturation that depends on the partial coordinate y as

$$\sigma = \sigma_+ \varsigma(y/d), \tag{10}$$

with $\varsigma(0) = 1$ and $\varsigma'(y) < 0$. We also impose that $\varsigma(\vartheta) = \sigma_-/\sigma_+ < 1$, where ϑ is a fixed number so that the supersaturation is equal to σ_- for $y = \vartheta d$. We would then like to evaluate the nucleation rate per unit length of facet edge in the direction x

$$J_x = \int_0^{\vartheta d} \mathrm{d}y\; J_c(\sigma)\,\mathrm{e}^{-\sigma_c/\sigma}. \tag{11}$$

Since we assume a monotonically decreasing supersaturation profile, we can change variables and integrate over $s = \sigma/\sigma_+$. In the presence of an essential singularity of the form $\mathrm{e}^{-b/s}$ when $s \to 0$, and for any function

$g(s)$ that is finite and regular at $s = 1$ (with possible algebraic divergence at $s \to 0$), we have:

$$\int_{\sigma_-/\sigma_+}^{1} \mathrm{d}s\; g(s)\,\mathrm{e}^{-b/s} \approx g(1)\,\frac{\mathrm{e}^{-b}}{b}. \tag{12}$$

when $b \gg 1$ and $b \gg 1/(1-\sigma_-/\sigma_+)$. Hence, under the conditions

$$\sigma_+ \ll \sigma_c \tag{13}$$
$$\sigma_+ - \sigma_- \gg \sigma_+^2/\sigma_c \tag{14}$$

the nucleation rate will be dominated by the behavior close to $y = 0$. Since the boundary condition $\varsigma(0) = 1$ implies $\varsigma^{-1}(1) = 0$, we obtain

$$J_x = \frac{d}{-\varsigma'(0)}\,\frac{\sigma_+}{\sigma_c}\,J_+ = \frac{1}{-\frac{\mathrm{d}\sigma}{\mathrm{d}y}|_{y=0}}\,\frac{\sigma_+^2}{\sigma_c}\,J_+ \tag{15}$$

where $J_+ = J|_{\sigma=\sigma_+}$.

A similar analysis also allows one to determine the typical distance y from the edge at which nucleation events should be observed for a given supersaturation profile:

$$\langle y\rangle_{nuc} = \frac{1}{J_x}\int_0^{\vartheta d} \mathrm{d}y\; y\,J_c(\sigma)\,\mathrm{e}^{-\sigma_c/\sigma}. \tag{16}$$

Using again the same strategy as for Eq.(12), but now with a function $g(s)$ that vanishes for $s = 1$, i.e. $g(1) = 0$, we have for $b \gg 1$:

$$\int_{\sigma_-/\sigma_+}^{1} \mathrm{d}s\; g(s)\,\mathrm{e}^{-b/s} \approx -g'(1)\,\frac{\mathrm{e}^{-b}}{b^2}. \tag{17}$$

leading to

$$\langle y\rangle_{nuc} = \frac{d}{-\varsigma'(0)}\,\frac{\sigma_+}{\sigma_c} = \frac{\sigma_+^2}{-\frac{\mathrm{d}\sigma}{\mathrm{d}y}|_{y=0}\sigma_c} \tag{18}$$

This leads to the simple formula

$$J_x = \langle y\rangle_{nuc} J_+. \tag{19}$$

When nucleation is confined along the edge of the facet, the total nucleation rate is equal to PJ_x where P is the perimeter of the facet edge. Since the rate of nucleation is constant as long as nucleation does not occur, the probability of having no nucleation event up to the time t is Poissonian:

$$\mathcal{P}(t) = \mathrm{e}^{-\int_0^t \mathrm{d}\tau P J_x} \tag{20}$$

Note that here, the time t starts at a conventional time $t = 0$ when the system is reset to a reference state. As a consequence, the probability density $Q(t)$ that the first nucleation occurs at time t is

$$Q(t) = -\partial_t \mathcal{P}(t). \tag{21}$$

The average time for nucleation to occur is then evaluated as

$$\langle t\rangle_{nuc} = \int_0^\infty \mathrm{d}t\; t\, Q(t) = \int_0^\infty \mathrm{d}t\; t\, P J_x \mathrm{e}^{-\int_0^t \mathrm{d}\tau P J_x}. \tag{22}$$

The expected value of the position of the nucleation event when it occurs is

$$\langle y\rangle_{loc} = \int_0^\infty \mathrm{d}t \int_0^\infty \mathrm{d}y\; y\,\frac{J(y,t)}{J_x(t)}Q(t) = \int_0^\infty \mathrm{d}t\,\frac{J_x(t)^2 P}{J_+}\mathrm{e}^{-\int_0^t d\tau P J_x(\tau)}. \tag{23}$$

We now apply Eqs.(23,22) to specific forms of the supersaturation profile.

a. **Localization with pre-existing steps on the facet.** As a first example, we aim to mimic a situation where a cavity is forming in the center of the facet. The steps at the edge of the cavity maintain the supersaturation to a lower value σ_-. Hence, d is constant, or varies slowly. Assuming a steady-state saturation profile, we obtain a linear decrease of σ from σ_+ at $y = 0$ to σ_- at $y = d$:

$$\sigma = \sigma_+ - (\sigma_+ - \sigma_-)\frac{y}{d} \tag{24}$$

We then have

$$\varsigma(u) = 1 - (1 - \frac{\sigma_-}{\sigma_+})u \tag{25}$$

and $\vartheta = 1$. As a consequence, $\varsigma'(u) = -(1 - \sigma_-/\sigma_+)$, leading to

$$J_x = \frac{d}{\sigma_+ - \sigma_-}\,\frac{\sigma_+^2}{\sigma_c}\,J_+ \tag{26}$$
$$\langle y\rangle_{nuc} = \frac{d}{\sigma_+ - \sigma_-}\,\frac{\sigma_+^2}{\sigma_c} \tag{27}$$

If d varies slowly in time (i.e. varies at a time-scale larger than the time between two nucleation events), then from Eq.(22), we have

$$\langle t\rangle_{nuc} = \frac{1}{PJ_x} \tag{28}$$

We also find average distance of the nucleation event

$$\langle y\rangle_{loc} = \langle y\rangle_{nuc} \tag{29}$$

As expected, the since the configuration is time-independent, the location of the first nucleation event $\langle y\rangle_{loc}$ is the same as the location $\langle y\rangle_{nuc}$ of a nucleation event that would occur if nucleation occurs at any arbitrary time t.

These results suggest that nucleation is still be localized after the formation of the cavity in the center of the facet. Assuming a bunch of steps at a distance of about $d = 50\mu$m from the edge creating a zone where $\sigma_- \approx 0$, and using $\sigma_+ \approx 0.05$ and $\sigma_c = 1$, we obtain $\langle y\rangle_{loc} \approx 2.5\mu$m. This is consistent with experimental observations.

b. **Diffusion-limited relaxation of the supersaturation in the absence of other steps.** As a second example, we consider a supersaturation profile that results from the relaxation of the supersaturation profile by diffusion after the passage of a constant velocity step. We assume that the initial state is a homogeneous supersaturation σ_- for all $y > 0$ at $t = 0$. At $y = 0$, the supersaturation is assumed to be constant and equal to $\sigma_+ > \sigma_-$ at all times. The solution of the diffusion problem provides:

$$\sigma = (\sigma_+ - \sigma_-)\mathrm{erfc}(y/(4Dt)^{1/2}) + \sigma_-. \quad (30)$$

This leads to

$$\varsigma(u) = (1 - \frac{\sigma_-}{\sigma_+})\mathrm{erfc}(u) + \frac{\sigma_-}{\sigma_+} \quad (31)$$

$$d = (4Dt)^{1/2} \quad (32)$$

and $\vartheta = \infty$, i.e. the supersaturation decreases to zero at $y \to \infty$. In order to use our model based on the nucleation rate (7), we need to assume that a steady-state for the distribution of monolayer island sizes explored by thermal fluctuations is reached with a time-scale that is faster than that of the evolution of the supersaturation. This assumption is not valid here, and our model therefore only provides a lower bound for the nucleation time. However, we expect our approach to catch the main features of the localization of nucleation.

Using equations (22,23), we now obtain:

$$J_x = (\pi Dt)^{1/2} \frac{\sigma_+^2}{(\sigma_+ - \sigma_-)\sigma_c} J_+. \quad (33)$$

$$\langle y \rangle_{nuc} = (\pi Dt)^{1/2} \frac{\sigma_+^2}{(\sigma_+ - \sigma_-)\sigma_c} \quad (34)$$

Assuming that the variation of the perimenter P and the supersaturation σ_+ and σ_- are slower than the variation of d, we find

$$\langle t \rangle_{nuc} = \frac{\Gamma[5/3]}{(\pi D)^{1/3}} \left(\frac{3}{2} \frac{(\sigma_+ - \sigma_-)\sigma_c}{\sigma_+^2 J_+ P} \right)^{2/3} \quad (35)$$

We have fitted to the experimental data in Figure 2 using the expressions (7) and (34) with σ_c and ρ_s as free parameters. We obtain $\Omega_2 \rho_s = 2.8 \times 10^{-6}$. In addition, we find $\sigma_c \approx 1.4$, which leads to $\Gamma \approx 0.44$ nm. These values are close to those obtained using the expression for a homogeneous nucleation rate on the facet.

We also obtain the position $\langle y \rangle_{loc}$ of the nucleation event as:

$$\langle y \rangle_{loc} = \left(\frac{\pi D}{J_+ P} \right)^{1/3} \left(\frac{\sigma_+^2}{(\sigma_+ - \sigma_-)\sigma_c} \right)^{2/3} \frac{3^{1/3}}{2^{1/3}} \Gamma[\frac{4}{3}] \quad (36)$$

Hence

$$\frac{\langle y \rangle_{loc}}{\langle y \rangle_{nuc}|_{t=t_{nuc}}} = \frac{\Gamma[\frac{4}{3}]}{\Gamma[\frac{5}{3}]^{1/2}} = 0.9398.. \quad (37)$$

Since this ratio is close to 1, we conclude that the detailed description of the time-dependence of the position of the nucleation event via the time-dependence of d does not bring a significiant quantitative correction to the average position of nucleation.

We can also rewrite the result as

$$\langle y \rangle_{loc} = (\pi D \langle t \rangle_{nuc})^{1/2} \frac{\sigma_+^2}{(\sigma_+ - \sigma_-)\sigma_c} \frac{\Gamma[\frac{4}{3}]}{\Gamma[\frac{5}{3}]^{1/2}} \quad (38)$$

Quantitatively, we find that d varies from 50μm in 1s to about 150μm in 10s. Assume that the supersaturation has been depleted by the passage of a step in a film of thickness 30nm. Then from Eq.(42), we obtain $\sigma_+ - \sigma_- = 1/\Theta_{eq} \approx 0.036$. Note that this difference is smaller, but of the same order as the supersaturation imposed at the boundary in the single-nucleation regime $\sigma_+ \approx 0.05$. We finally obtain from Eq.(38) $\langle y \rangle_{loc} = 3\mu$m for a nucleation time $\langle t \rangle_{nuc} \approx 1$s, and $\langle y \rangle_{loc} = 9\mu$m for a nucleation time $\langle t \rangle_{nuc} \approx 10$s.

Step front instability

Experiments

Several experiments showed unstable step fronts. In most cases the crystals were too small to show well developed fingers that lend themselves to quantitative analysis. We therefore concentrate the discussion on the experiment shown in movie S4 and Figs. 4 and S5 where the bulk supersaturation is 0.053 and the $\bar{\zeta} = 20$ nm. The first four consecutive step fronts pass at time intervals of 54±7 s and develop finger-like instabilities. All 4 unstable fronts travel in a direction between the two maximum kinetic anisotropy directions (see arrows of kinetic anisotropy in Fig. 4 A-D). The fifth step front (Fig. S5 D) arrives only 9 seconds after the preceeding front and travels in the low velocity direction. The time between fronts and the direction of travel are therefore important to whether the fronts destabilize or not.

The time t between two fronts determines how far diffusion has transported ions along the confined crystal surface. The diffusion length $l = \sqrt(Dt)$ is 180 μm for the unstable fronts and 70 μm for the last front. No part of the images are more than 160 μm from a crystal edge. The fact that the last front starts out with a velocity of 18 μm/s and slows down with the square root of time is consistent with the front moving in a diffusion controlled concentration field.

Fig. 4A-C follow the progression of one of the unstable fronts. In Fig. S5 B the finger tips are indicated with red circles and the slow part of the front is indicated with a dashed green line. The finger tips propagate at constant speed and the slow part propagates at a velocity slowing down with the square root of time. This indicates that the fingers propagate by using the ions already present in the confined fluid film whereas the slow part depends on

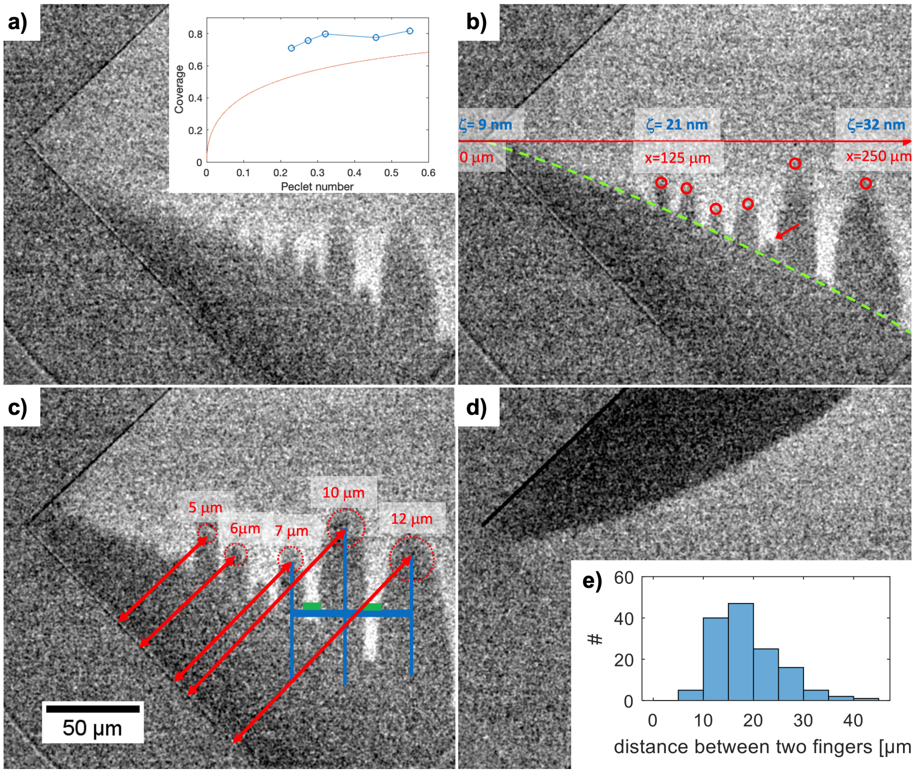

FIG. S5. Instability of step flow. **a)-c)** consecutive average subtracted RICM images at 0.45 s interval of step flow at an edge of a confined $NaClO_3$ interface showing instability of the front. The dark areas correspond to a smaller distance to the confining glass and thus to the newly formed layer. Tracking of tips and step front base is indicated in b). The crystal is tilted along the red line in b), the glass-crystal distances ζ are given in blue for the distances (in red) along the red arrow. In c) we indicate the measurements of finger tip radii, where the distance to the crystal edge is measured (red arrows) and the measurement of distance between finger midpoints (blue lines) and width of zone that is not covered by the fast, instable front (green). d) Slow double layer front at the same interface and under the same conditions directly after a)-c) not showing instabilities. e) Histogram of distance of adjacent fingers with a mean value of 18.6 nm and a standard deviation of 6.4 nm. Frame rate: $1/dt = 1/450$ ms, integration time: $t_{ac} = 450$ ms, supersaturation in the bulk: $\sigma = 0.053$. Inset in a) is a comparison between the theoretical relation between Peclet number and coverage from equation(46) and the corresponding parameters from measurements in these images.

diffusion transport of ions from the bulk to propagate. The fraction of the area covered by the fingers before the slow front arrives is 0.7-0.9. One interpretation of this finite coverage by the fast growing layer is that it is limited by the number of ions already present in the fluid film. The ion coverage in the confined fluid is $\Theta_{eq}\sigma = \zeta c_0\sigma/(z_0 c_s) = \zeta\sigma/1.2$ nm, where $c_0/c_s = 0.72/2.54$ and $z_0 = 0.33$ nm.

Theoretical considerations

Let us consider a straight and isolated atomic step along the x axis moving at velocity V in the y direction during growth. If small perturbations $\xi(x,t)$ of the step position along y are amplified, then the step is unstable. When the perturbation is small enough, the dynamical equations governing the motion of the step can be analyzed in perturbations, and to leading order one obtains a set of linear equations governing the evolution of $\xi(x,t)$. Assuming for simplicity that the step velocity is constant in time, a single Fourier mode of the perturbation $\xi_q(t)$ of wavevector q therefore grows or decays exponentially in time as $e^{i\omega t}$. The linear equations for $\xi_q(t)$ then provide a relation between $i\omega$ and q, which is called the dispersion relation. If $\Re e[i\omega] > 0$ for some values of q, then the perturbations $\xi_q(t)$ will grow exponentially in time, and the step will be unstable.

In the long wavelength limit, the dispersion relation contains two terms. The first term is destabilizing and is proportional to the velocity V, and the second term is a stabilization term due to line tension effects

$$i\omega = Vq - 2\tilde{\Gamma}\Theta_{eq}Dq^3 \tag{39}$$

where D is a diffusion constant, and $\tilde{\Gamma} = \Omega\tilde{\gamma}/nk_BT$ is the so-called capillary length, where Ω is the molecular area in a solid layer, $n = 2$ is the number of ions in the liquid for one solid molecule, k_BT is the thermal energy and $\tilde{\gamma}$ is the step stiffness. The stiffness is in general a function of the orientation angle ϕ, and is related to the

step free energy $\gamma(\theta)$ via the relation $\tilde{\gamma}(\theta) = \gamma(\theta)+\gamma''(\theta)$.

From the condition $i\omega > 0$, we find that the perturbations with wavelength larger than

$$\lambda_0 = 2\pi(2\tilde{\Gamma}\Theta_{eq}\ell)^{1/2}, \quad (40)$$

where $\ell = D/V$ is usually called the diffusion length, are unstable. The wavelength of the most unstable mode, i.e., the mode with the largest $\Re e[i\omega]$, reads

$$\lambda_m = 3^{1/2}\lambda_0 = 2\pi(6\tilde{\Gamma}\Theta_{eq}\ell)^{1/2}. \quad (41)$$

In the isotropic case, we have $\tilde{\gamma} = \gamma$ and $\tilde{\Gamma} = \Gamma/n$, leading to the expression of the main text.

When the step is unstable, small perturbations grow until nonlinearities come into play. These nonlinearities control the emerging morphology. A number of studies have focused on similar problems in two or three dimensions.

The driving force is measured by the coverage $\Theta_{eq}\sigma = \zeta c_0\sigma/(z_0 c_s) = \zeta\sigma/1.2$ nm, where $c_0/c_s = 0.72/2.54$, c_s is the molar density of the solid, ζ is the liquid film thickness and z_0 is the height of a monolayer. This number measures the number of monolayers of solid that can be formed from the excess of ions in the supersaturated liquid. Considering a supersaturation $\sigma \approx 0.05$, we obtain $\Theta_{eq}\sigma \approx \zeta/22$ 1/nm where the film thickness ζ is in nanometers.

If $\Theta_{eq}\sigma > 1$, i.e. in our experiments if $\zeta > 22$nm, there is enough material in the liquid for a straight step to grow at constant velocity into a supersaturation σ. The supersaturation decreases from the value σ far in front of the step to the value $\sigma_<$ at the step and behind it, which is depleted by an amount that corresponds to the formation of the new monolayer:

$$\sigma_< = \sigma - \frac{1}{\Theta_{eq}} \quad (42)$$

The step velocity then reads

$$V = \alpha k(\theta)\sigma_< \quad (43)$$

where the kinetic coefficient $\alpha k(\theta)$ is defined in the main text.

Linear stability analysis suggest that these steps undergo a Mullins-Sekerka instability when

$$\Theta_{eq}\sigma < 1 + \frac{D}{\alpha k(\theta)\tilde{\Gamma}} \quad (44)$$

The nonlinear dynamics has only been investigated theoretically close to the threshold [5], suggesting that isotropic steps undergo spatio-temporal chaos governed by the Kuramoto-Sivashinsky equation. However, in our experiments $\Theta_{eq}\sigma$ is at most ~ 5 while $nD/(\bar{\alpha}\Gamma) \sim 10^3$. This correspond to a regime which is far from the threshold, where $q_0\ell \gg 1$, with $q_0 = 2\pi/\lambda_0$. In this limit, the usual expression (40) of λ_0 is valid, but to our knowledge, nothing is known about nonlinear dynamics.

In the other regime when the coverage is low $\Theta_{eq}\sigma < 1$, corresponding to small film thicknesses $\zeta < 1.2/\sigma$ nm, there is not enough ions in the liquid film to form a monolayer via the motion of a step. As a consequence, ions have to diffuse from an increasing distance to be incorporated in the step as time goes forward. This results in a decreasing step velocity $v_{step} \sim (D/t)^{1/2}$.

This regime gives rise to the celebrated "dendrite" morphologies. Dendrites correspond to a constant-velocity parabolic step profile, which possibly undergoes side-branching away from the tip. The growth direction and the tip radius are controlled by anisotropy. In contrast, when anisotropy is negligible, one finds seaweed shapes characterized by permanent branching due to the splitting of their tips.

Irrespective of anisotropy, the tip of the parabolic Ivanstov dendrite solution [6] is constrained by a relation between the tip radius R and the tip velocity v_{tip}:

$$p = \frac{Rv_{tip}}{2D}. \quad (45)$$

where p is a dimensionless number called the Peclet number, which exhibits a non-trivial dependence on the coverage

$$\Theta_{eq}\sigma = (\pi p)^{1/2}\mathrm{e}^{p}\mathrm{Erfc}[p^{1/2}] \quad (46)$$

Inset in Fig. S5 a) is a comparison between this relation between Peclet number and coverage and the corresponding parameters from measurements. In Fig. S5 we indicate the measurements or R for each of the fingers. Using $v_{tip} = 55\mu$m and $D = 0.6 \cdot 10^{-9}$m^2/s we obtain the experimental Peclet numbers p from equation (45).

The time scale of motion of the fingers is short compared to the long time scale of the diffusion controlled evolution of supersaturation in the thin film. We may estimate the coverage $\Theta_{eq}\sigma$ at the time of passage of the fingers in two different ways: by solving the diffusion equation or by measuring the fraction of the area covered by the fingers.

Because the crystal is tilted and because of the finite time since the last layer grew the coverage at each finger tip is a function of its position. The supersaturation can be estimated as a function of distance to the nearest edge (as indicated in Fig S5) and the time t since the last growth front passed:

$$\sigma(x,t)/\sigma(x=0) = \mathrm{erfc}\left(\frac{x}{2\sqrt{(Dt)}}\right). \quad (47)$$

This assumes that $\sigma(t=0) = 0$ and is not corrected for the tilt of the crystal. In order to calculate the coverage $\Theta_{eq}\sigma = \zeta\sigma/(1.2 \text{ nm})$ the glass-crystal distance ζ is evaluated for each tip. The resulting coverages in the range 0.7-0.8 are plotted in Fig.S5 a. For one of the fingers we may confidently estimate the coverage by the fraction of the crystal area the fingers cover before the slow front arrives. Fig. S5 c shows this measurement of coverage

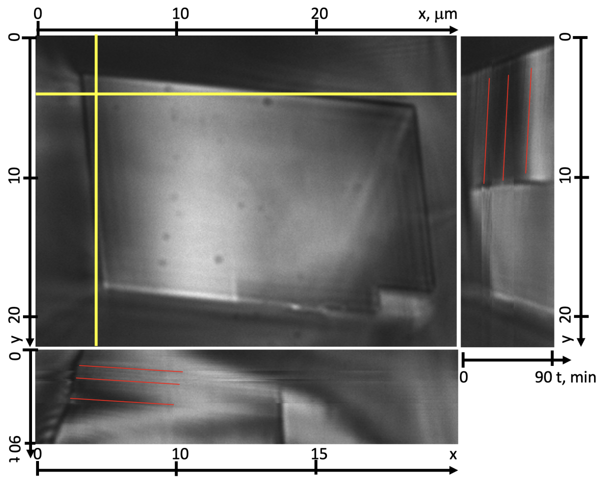

FIG. S6. Surface of calcite crystal 30 nm above confining glass surface in water with 0.8 mM $CaCO_3$ concentration ($\sigma = 0.6$) imaged once every minute. Bottom and right plot show intensities along yellow line as function of time. The red lines trace the motion of waves of step flow during growth of the confined calcite surface. The slope of the red lines yield the step flow velocity in the x- and y-direction.

$\Theta_{eq}\sigma \approx 0.7$ for the second rightmost finger. This is 10% smaller than estimated from the diffusion calculation. In the inset of Fig. S5 a) we plot the coverage (from the diffusion calculation) for each finger tip as function of Peclet number from (45). This agrees very well with the prediction from equation (46).

Two-dimensional numerical simulations of the dynamics with coverage lower than one has been reported in many papers (see for example [7]). The results show dendrites and seaweed features at small values of the coverage. However, when the coverage approaches 1, a continuous front emerges with a porous monolayer. As a consequence, fingering should actually only be observed approximatively for $\Theta_{eq}\sigma < 0.9$. The porosity of the newly formed monolayer decreases as one approaches unit coverage.

CALCITE EXPERIMENTS

The calcite experimental setup has been described in detail elsewhere [8]. A calcite crystal (see Figure S6) of approximately 15x25 μm is kept at a $CaCO_3$ concentration of 0.801±0.002 mM, which corresponds to a supersaturation of $\sigma = 0.6$ and a saturation index of Ω=0.44 [9].

Supplementary movie SM6 shows "waves" of high and low intensity move along the rim from bottom left, around the top left corner and on to the top right. These intensity variations are due to changes in the glass-crystal distance ζ as molecular steps move across the surface. The step flow emanates from a step edge on the rim in much the same way as from the dislocation source in Figure 2.

From [8] we know that when the distance between the glass surface and the calcite surface was 30 nm the vertical growth rate was 2.6 nm/min. The step height on calcite is 0.34 nm and the vertical growth rate of 8 layers per minute corresponds to a horizontal single molecular step spacing of 170-340 nm. This is well below the horizontal resolution limit of the objective in this experiment and the waves of step flow observed in movie SM6 correspond to several molecular steps, either equidistant or joined in step bunches. The growth regime on this calcite surface is not a "single step" regime as for several of the $NaClO_3$ experiments. Thus, even though we cannot resolve single steps, the interference contrast allows us to measure the "collective" speed of the molecular steps along the surface.

Figure S6 shows the same crystal at time $t = 0$ with a vertical and a horizontal yellow line that indicate where intensity has been measured and displayed as function of time in the two side images. The motion of the intensity change caused by the flow of molecular steps manifests as dark and bright lines in the $x - t$ and $y - t$ images in Figure S6 and the slope of the lines measure the velocity of the molecular steps. We have highlighted some of these lines with red in Figure S6. The step flow velocities are determined to be 43±10 nm/s in the x-direction and 22±5 nm/s in the y-direction. This is one order of magnitude faster than determined from AFM measurements[10, 11] at similar reported saturation index. We have, however shown that calcite growth rates as function of saturation index measured by AFM are 2 orders of magnitude too small, probably due to insufficient control of the saturation index at the calcite surface [12]. Since the supersaturation at the confined surface decreases with distance from the crystal edge we find our present measurement $v_s/\sigma = 40 - 70$ nm/s in agreement with previous findings.

USING TEMPERATURE TO CONTROL SUPERSATURATION

There is a long tradition for using temperature to control supersaturation in crystal growth studies for NaClO3 and many other crystals. For example, temperature differences of up to 5 K were used to control supersaturation in a study of the growth kinetics of $NaClO_3$ without showing any visible effect on the growth rate [13]. The largest supersaturation in our study (the experiments showing step flow instability) corresponds to a temperature change no larger than 10 K. Such a temperature change changes the self diffusion coefficient of water by about 25% and we expect the step flow velocity to change by a similar magnitude. The cavity formation has been shown for calcite where we do not use temperature for supersaturation control. The step flow instability depends on the ratio v_s/D which more or less cancels the temperature effects on v_s and D. We

therefore have no reason to assume that there are dramatic changes in the kinetic parameters that affect the nature of our results.

[1] Israelachvili, J. N. *Intermolecular and surface forces* (Associated Press, Oxford, 2011), 3rd edn.

[2] Mutisya, S. M., Kirch, A., De Almeida, J. M., Sánchez, V. M. & Miranda, C. R. Molecular Dynamics Simulations of Water Confined in Calcite Slit Pores: An NMR Spin Relaxation and Hydrogen Bond Analysis. *Journal of Physical Chemistry C* **121**, 6674–6684 (2017).

[3] Smith, A. M., Lee, A. A. & Perkin, S. The Electrostatic Screening Length in Concentrated Electrolytes Increases with Concentration. *Journal of Physical Chemistry Letters* **7**, 2157–2163 (2016).

[4] Saito, Y. *Statistical Physics of Crystal Growth* (World Scientific, Singapore, 1996).

[5] Misbah, C., Pierre-Louis, O. & Saito, Y. Crystal surfaces in and out of equilibrium: A modern view. *Reviews of Modern Physics* **82**, 981–1040 (2010).

[6] Kassner, K. *Pattern Formation in Diffusion-Limited Crystal Growth* (World Scientific, Singapore, 1996).

[7] Ben-Jacob, E. From snowflake formation to growth of bacterial colonies. *Contemporary Physics* **34**, 247–273 (1993).

[8] Li, L. *et al.* Limits to crystallization pressure. *Langmuir* **38** 11265-11273 (2021).

[9] Li, L., Kohler, F., Røyne, A. & Dysthe, D. Growth of Calcite in Confinement. *Crystals* **7**, 361 (2017).

[10] Teng, H. H., Dove, P. M. & De Yoreo, J. J. Kinetics of calcite growth: Surface processes and relationships to macroscopic rate laws. *Geochimica et Cosmochimica Acta* **64**, 2255–2266 (2000).

[11] Bracco, J. N., Stack, A. G. & Steefel, C. I. Upscaling calcite growth rates from the mesoscale to the macroscale. *Environmental Science and Technology* **47**, 7555–7562 (2013).

[12] Li, L., Sanchez, J. R., Kohler, F., Røyne, A. & Dysthe, D. K. Microfluidic Control of Nucleation and Growth of CaCO3. *Crystal Growth and Design* **18**, 4528–4535 (2018). 1708.06299.

[13] Ristic, R., Sherwood, J. N. & Wojciechowskf, K. Morphology and Growth Kinetics of Large Sodium Chlorate Crystals Grown in the Presence and Absence of Sodium Dithionate Impurity. *J. Phys. Chem* **97**, 10774–10782 (1993).
