# Supplementary material for: Crystal growth in confinement
Source: Nat Commun. 2022 Nov 16;13:6990. doi: 10.1038/s41467-022-34330-5 (PMC9669051; doi:10.1038/s41467-022-34330-5)
Supplement: Supplementary file 2 — Description of Additional Supplementary Files [file 41467_2022_34330_MOESM2_ESM.pdf]

Description of Additional Supplementary Files

Movie captions for Crystal growth in confinement

Felix Kohler,[1] Olivier Pierre-Louis,[2] and Dag Kristian Dysthe[1]

[1] *The NJORD Centre, Dept of Physics, University of Oslo, P.O.box 1048 Blindern, 0316 Oslo, Norway*

[2] *Institut Lumière Matière, UMR5306 Université Lyon 1 - CNRS, 69622 Villeurbanne, France*

MOVIES

The supplementary movies are available on figshare with the following URL: https://doi.org/10.6084/m9.figshare.21201737

Supplementary Movie 1

Single molecular layers nucleated and propagating over crystal surface at low supersaturation ($\sigma = 0.051$) and low nucleation rate. Timelapse movie of average subtracted RICM images at 0.1 s interval. The dark areas correspond to a smaller distance to the confining glass and thus to the newly formed single molecular layer 0.33 nm thick. The four nucleation events originate at different locations. The crystal is $160 \times 160 \mu$m.

Supplementary Movie 2

Single atomic layers nucleated and propagating over crystal surface creating concentration gradient and tending towards cavity formation. Timelapse movie of average subtracted RICM images at 0.1 s interval. The dark areas correspond to a smaller distance to the confining glass and thus to the newly formed single molecular layer 0.33 nm thick. Same crystal as in Supplementary Movie 1 and Fig. S4 with $\sigma = 0.055$. Due to higher nucleation rate than in Supplementary Movie 1 the diffusion of ions does not replenish ion concentration at the centre resulting in a concentration gradient from edge to centre. Due to the concentration gradient nucleation centres are located at the crystal edge and the molecular layer step flow is slower towards the centre than along the edges. Fluctuations in nucleation frequency causes fluctuations in concentration gradient and stability of cavity.

Supplementary Movie 3

Fluctuations in nucleation and cavity formation. Timelapse movie of average subtracted RICM images at 1 min interval. Same crystal as in Supplementary Movies 1 and 2 and Fig. S4 A and B at $\sigma = 0.06$. The crystal size increases during the movie thus increasing the diffusion time and the criterium for cavity formation is fulfilled: $\Theta_{eq}\sigma\tau_N/\tau_D < 1$. Fluctuations in nucleation frequency causes fluctuations in the stability of the cavity. A cross-section versus time of this movie is displayed in Fig S4.

Supplementary Movie 4

Step front instability. Timelapse movie of average subtracted RICM images of corner of a 700x700 μm^2 large crystal with σ=0.053 and $\bar{\zeta}$=22 nm. The dark areas correspond to a smaller distance to the confining glass and thus to the newly formed single molecular layer 0.33 nm thick, showing instabilities at the front. The four instable fronts propagate in the fast direction and display fingers that do not cover the entire surface move at constant velocity while a slow (and slowing down), diffusion controlled front fills the layer between the fingers. Some images from this movie are shown in Figs. 4 and S5.

Supplementary Movie 5

Spiral growth on a nanoconfined growth rim. Timelapse movie of average subtracted RICM images at 1 s interval. The oval regions of different intensity are molecular layers 0.33 nm in height each. Images in this movie are also displayed in Fig. 3. The top and right hand side of the movie is the crystal edge and the grey area at bottom left is the cavity inside the growth rim. One observes that every second layer has different propagation velocity and shape due to the different (rotated 180 degrees) kinetic anisotropy ratios of the two half layers constituting a unit cell of the crystal.

Supplementary Movie 6

Corner of calcite crystal 30 nm above confining glass surface in water with 0.8 mM $CaCO_3$ concentration ($\sigma = 0.6$) imaged once every minute for 90 minutes. The waves moving from bottom left to top right are interference contrast of growth steps propagating on the confined surface.
